# Z-Ligustilide Induces c-Myc-Dependent Apoptosis *via* Activation of ER-Stress Signaling in Hypoxic Oral Cancer Cells

**DOI:** 10.3389/fonc.2022.824043

**Published:** 2022-04-13

**Authors:** Ren-Jun Hsu, Kui-Yuan Peng, Wen-Lin Hsu, Yu-Tang Chen, Dai-Wei Liu

**Affiliations:** ^1^ Cancer Center, Hualien Tzu Chi Hospital, Buddhist Tzu Chi Medical Foundation, Hualien, Taiwan; ^2^ School of Medicine, Tzu Chi University, Hualien, Taiwan; ^3^ Institute of Medical Sciences, Tzu Chi University, Hualien, Taiwan; ^4^ Department of Radiation Oncology, Hualien Tzu Chi Hospital, Buddhist Tzu Chi Medical Foundation, Hualien, Taiwan

**Keywords:** Z-ligustilide (LIG), c-Myc, oral cancer cells, radiosensitizer, ER stress

## Abstract

Z-ligustilide (or ligustilide) is found in *Angelica sinensis* (*Oliv.*) *Diels* and may exert potential benefits in cancer treatment. Previous research has reported that ligustilide has anti-cancer effects on several types of cancer cells. However, studies of ligustilide on oral cancer cells have not been reported, especially under hypoxic conditions. This study focuses on the molecular mechanism of ligustilide-induced apoptosis in hypoxic oral cancer cells. We found that in hypoxic TW2.6 cells, ligustilide inhibited cell migration and induced caspase-dependent apoptosis. Accumulation of c-Myc accompanied by BH3-only members suggests that ligustilide may induce c-Myc-dependent apoptosis. In addition, we reported that ligustilide has an effect on ER-stress signaling. By using inhibitors of c-Myc, IRE1α, and ER-stress inhibitors, we found that cell morphologies or cell viability were rescued to some degree. Moreover, ligustilide is able to increase the expression of γ-H2AX and enhance the occurrence of DNA damage in oral cancer cells after radiation treatment. This result suggests that ligustilide has potential as a radiation sensitizer. Altogether, we propose that ligustilide may induce c-Myc-dependent apoptosis *via* ER-stress signaling in hypoxic oral cancer cells.

## Introduction

Z-ligustilide (or ligustilide) is one of the two major bioactive components isolated from *Angelica sinensis* (*Oliv.*) *Diels* (Danggui), along with n-butylidenephthalide (1). Research has shown that ligustilide exerts therapeutic potential in several diseases including Alzheimer’s disease, diabetic nephropathy, osteoarthritis, ovarian cancer, and breast cancer (2). Despite reported inhibitory effects on other types of cancer cells, the mechanism underlying these effects is not yet clear (3–6). It has been shown that ligustilide restored NRF2 expression in ovarian cancer cells and epigenetic control associated with apoptosis. Further evidence suggests that NRF2 promotes cell survival against ligustilide-induced oxidative stress (7). In tamoxifen-resistant breast cancer cells, ligustilide resensitized cells by inhibiting autophagy through the restoration of Nur77 expression, which blocks DNA repair induced by tamoxifen (8). Another study suggested that ligustilide resensitized ERα negative breast cancer cells *via* epigenetic restoration of ERα through HDACs and other factors (9). Overall, ligustilide may elicit an epigenetic regulatory response to attenuate cancer survival or resistance in ovarian and breast cancer cells.

Oral cancer is included in the category of head and neck squamous cell carcinoma (HNSCC) and is one of the most common types of cancer worldwide (10). Most types of oral cancer are oral squamous cell carcinomas, with the following key risk factors: alcohol consumption, cigarette smoking, and betel nut chewing, as well as infection with human papillomavirus (HPV). More than 70% of patients with oral cancers in the early stages can be cured by surgery or radiation therapy (11). However, nearly 50% of oral cancer patients in Taiwan are not diagnosed in the early stages, which results in a survival rate of less than 50% in 5 years, following intervention for patients with stage III or IV cancer (12, 13). The main obstacles to treatment are local recurrence of tumors and metastasis (14).

Combination therapy refers to a combination of two or more therapies that typically contain one traditional therapy that has been found to improve the effectiveness of cancer treatment. Natural compounds from traditional herbal medicine such as curcumin or ligustilide have the potential to improve the efficacy of cancer treatment (2, 15). Thus, it is imperative to explore the molecular mechanisms of natural compounds and their effects on cancer.

Hypoxia is considered a hallmark of cancer-promoting tumor progression (16). Hypoxia-inducible factor 1α (HIF-1α) is a key transcriptional factor that initiates this adaptative process through its accumulated effects on protein levels under hypoxic conditions. HIF-1α protein is believed to be controlled mainly *via* a post-translational pathway. Low oxygen tension attenuates phosphorylation of HIF-1α by prolyl hydroxylases (PHDs) and therefore blocks von Hippel Lindau (VHL)-mediated ubiquitination and degradation. Hypoxia is experienced not only in malignant tumors but in normal tissues as well. Specifically, 2% to 9% O_2_ is considered physiological hypoxia and represents a normal state for most organs (17). An oxygen level of less than 1% is generally considered as hypoxia in solid tumors. Overall, hypoxia not only mediates tumor adaptation but also is involved in normal tissue function.

c-Myc is a notorious proto-oncogene that is upregulated in a large portion of cancers (18). As a transcription factor, c-Myc affects gene expression in an estimated 15% of total genes. Target genes of c-Myc take part in cell cycle regulation, cell survival, protein synthesis, metabolism, and also microRNA expression. Thus, dysregulation of c-Myc is often associated with uncontrolled proliferation, transformation, or even cancer. However, c-Myc has shown an ability not only to exert pro-survival roles but also as a pro-apoptotic inducer. This intriguing scenario may partly be due to its downstream targets involving both pro-apoptotic and anti-proteins such as Bax, Bak, BH3-only members, and the Bcl2 family. The interacting partners of c-Myc such as Max, Mad, and Mxi1, and Max-interacting proteins may also be a factor that influences the outcome. Collectively, c-Myc exerts a complex network of interactions on gene regulation.

The endoplasmic reticulum (ER) is the main cellular organelle governing protein synthesis, folding, and delivery to the Golgi apparatus. Any dysregulation of normal ER function may lead to tipping the balance of protein homeostasis, which eventually elicits the unfolded protein response (UPR) (19). The UPR is well conserved in eukaryotes that deal with ER stress to maintain cellular homeostasis but execute programmed cell death when the level of stress is overwhelming. There are three sensors capable of receiving and firing the UPR, including inositol-requiring enzyme 1 (IRE1), activating transcription factor 6 (ATF6), and protein kinase RNA-like ER kinase (PERK). Each of these sensors has a unique function in mediating the UPR. For example, PERK can be activated by a variety of stressors, such as hypoxia and oxidative stress and directly lead to phosphorylation of the eukaryotic initiation factor 2-alpha (EIF2α). Consequently, global protein synthesis is reduced temporarily, but enhanced in certain genes such as ATF4 and CHOP. These transcriptional factors and other factors work together to determine cell fate *via* transcriptional, translational, and post-translational processes. To date, it is still not clear how translation selectivity is determined. Activation of the UPR could be either pro-survival or pro-apoptotic. It is commonly believed that mild stress induces the UPR to survive but promotes apoptosis when the stress exceeds its capacity. However, the fine-tuning of this process is also not clear.

First, although ligustilide exerts an anti-cancer effect on prostate cancer (4), non-small cell linear cancer (6), ovarian cancer (7), breast cancer (8), and other cancer cells, the exact mechanism involved has yet to be elucidated. The latest research suggests that ligustilide may inhibit the activation and apoptosis of cancer-associated fibroblasts (CAFs) in the tumor microenvironment, thereby altering the immunosuppressive function of CAFs (20). In addition, no research to date has suggested that ligustilide had anti-cancer effects in oral cancer cells, but it is known that a hypoxic microenvironment is a significant feature of oral cancer (21). Therefore, this study aims to explore the anti-cancer activity and related molecular mechanisms of ligustilide on hypoxic oral cancer cells.

## Materials and Methods

### Cell Culture, Hypoxia, Antibodies, and Chemicals

Human oral cancer cells TW2.6 (22) and OML1 (23) were described previously and obtained from Dr. Jeng-Woei Lee and Dr. Dai-Wei Liu, respectively. SCC-25 was purchased from Bio-resource Collection and Research Center (BCRC, Food Industry Research and Development Institute, Hsinchu, Taiwan). TW2.6 and OML1 were maintained in DMEM/F-12 supplemented with 10% FBS at 37°C in a humidified incubator containing 5% CO_2_. OML1 was maintained similarly but in RPMI medium supplemented with 10% FBS. Hypoxia was executed by a hypoxia incubator that maintained 1% O_2_ under stable conditions for at least 1 h (Astec Bio Corporation Ltd., Kasuya, Fukuoka, Japan). Antibodies were purchased from Abcam (Abcam, Cambridge, UK), Cell Signaling Technology (Danvers, Massachusetts, USA), and Genetex Corporation (Irvine, California, USA). Chemicals such as ligustilide or inhibitors were obtained from Sigma-Aldrich Corporation or Abcam. The pan-caspases inhibitor Q-VD-OPh and cisplatin were obtained from Genetex Corporation (Irvine, California, USA). Primary antibodies for HIF-1α, phospho-IRE1α, PARP-1, cyclin D1, and ATF4 were purchased from Genetex. Antibodies for IRE1α and CHOP/GADD153 were from Santa Cruz Biotechnology Incorporation (Santa Cruz, California, USA). Secondary antibodies were purchased from Abcam (Cambridge, UK). Other antibodies not mentioned here were purchased from Cell Signaling Technology (Danvers, Massachusetts, USA).

### MTT Assay

Cell viability was evaluated with thiazolyl blue tetrazolium bromide, and the MTT assay was carried out as described in the product information sheet (M2128, Sigma-Aldrich Corporation). In brief, cells (10.5 × 10^4^/cm^2^) were plated in 48-well plates overnight. Depending on the experiment, a medium containing MTT (0.5 mg/ml) was added as the final step. After 2 h of incubation at 37°C, the medium was carefully aspirated and DMSO was added to resolve formazan. Cell viability was measured with an optical density at 570 nm and referenced with O.D. 630 nm by an ELISA plate reader (μQuant, Biotek Instruments, Winooski, Vermont, USA).

### Western Blot, Pulse-Chase Assay, Immunoprecipitation, RNA Extraction, and Quantitative Real-Time PCR

Western blot was performed with a Bio-Rad system according to the instructions of Bio-Rad Laboratories (Hercules, California, USA). Briefly, cells were harvested with RIPA lysis buffer (Pierce Biotechnology, Waltham, Massachusetts, USA) supplemented with protease and phosphatase inhibitors (Thermo Fisher Scientific Incorporation, Waltham, Massachusetts, USA). Protein concentration was determined by the Lowry method (DC protein assay, Bio-Rad, Hercules, California, USA). Samples were mixed with an equal volume of 2× Laemmli sample buffer and denatured on a heat block. Proteins were separated by SDS-PAGE and transferred to PVDF membranes. After BSA blocking, primary antibodies were applied and incubated overnight on an incline shaker at 4°C. Membranes were washed with TBST, and secondary antibodies conjugated with HRP were applied for the appropriate time. After washing thoroughly with TBST, membranes were developed with ECL chemiluminescent substrate. Signal was captured by a detecting system equipped with a CCD camera (Wealtec Corporation, Sparks, Nevada, USA). ImageJ was used for further quantification.

Immunoprecipitation was performed using the PureProteome™ Protein G Magnetic Bead System (LSKMAGG02, Millipore). Quantified cell lysate (50 µg in 200 µl) was added to each reaction, and 4 µl of c-Myc (ab32071, abcam) was added to each reaction.

RNA for Q-PCR was extracted by an RNA purification kit according to its instructions (Item No. 12183018a, Invitrogen, Carlsbad, California, USA). RNA was subjected to first-strand cDNA synthesis by a cDNA synthesis kit (Item No. 04 379 012 001, Roche, Basel, Switzerland), and was subjected to Q-PCR by using the SYBR Green Master mix coupled with a Q-PCR reactor (Applied Biosystems, Foster City, California, USA). Messenger RNA levels of HIF-1α and VEGF-A were calculated by the comparative method referenced by solvent group and actin. Primers for individual genes are listed below:

HIF-1α: Forward: 5’-TGAGGAAATGAGAGAAATGCTTACA-3’, Reverse: 5’-ACACTGAGGTTGGTTACTGTTGGT-3’;

c-Myc: Forward: 5’-AGCGACTCTGAGGAGGAACA-3’, Reverse: 5’-CTCTGACCTTTTGCCAGG-3’;

NOXA: Forward: 5’-CGGAGATGCCTGGGAAGAA-3’; Reverse: 5’-CCAAATCTCCTGAGTTGAGTAGCA-3’;

PUMA: Forward: 5’-CTGTGAATCCTGTGCTCTGC-3’, Reverse: 5’-AATGAATGCCAGTGGTCACA-3’;

BIM: Forward: 5’-CAGCACCCATGAGTTGTGAC-3’, Reverse: 5’-TCTTGGGCGATCCATATCTC-3’;

XBP1s: Forward: 5’-GCTTGTGATTGAGAACCAGG-3’, Reverse: 5’-GCACCTGCTGCGGACTC-3’;

GAPDH: Forward: 5’-AAGGTGAAGGTCGGAGTCAA-3’, Reverse: 5’-AATGAAGGGGTCATTGATGG-3’.

### Wound-Healing Assay

Cell migration was analyzed by a wound-healing assay. Two-well culture inserts (ibidi, Gräfelfing, Bavaria, Germany) were set on 12-well plates before seeding cells. 10 × 10^4^ cells were plated in each well of the insert for 48 h. Culture inserts were removed, washed by PBS, and prepared for osthole stimulation. Subsequently, cells were incubated for 24 h under hypoxic conditions. Cell migration was calculated by the area of wound closure (%). Wound closure (%) = {[(cell free area)t_0_ – (cell free area)t_1_]_experiment_/[(cell free area)t_o_ – (cell free area)t_1_]_control_} × 100%.

### Statistical Analysis

All experiments were conducted independently at least three times. The data are presented as the mean ± SEM. Student’s *t*-test and one-way analysis of variance (one-way ANOVA) coupled with Scheffe’s post-hoc test was used to determine the significance.

## Results

### Ligustilide-Induced Caspase-Dependent Apoptosis and Inhibited Cell Migration in Oral Cancer Cells

**Figure 1:**
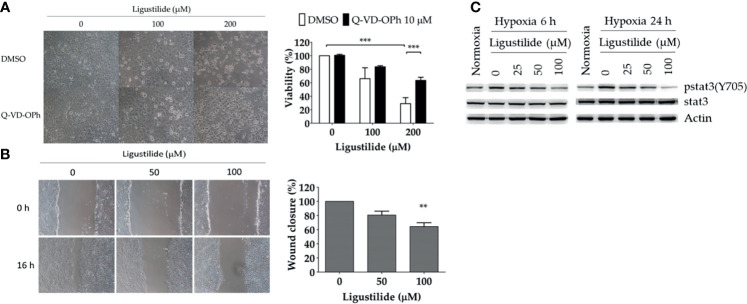
Ligustilide-induced caspase-dependent apoptosis and inhibited cell migration in oral cancer cells. **(A)** TW2.6 cells were pretreated with the pan-caspase inhibitor Q-VD-OPh (10 μM) for 30 min and incubated with ligustilide for another 24 h of hypoxia at 37°C. Cell viability was measured by MTT assay and images were captured and presented in 200 times magnification. Data in the plot are expressed as mean ± SEM (*n* ≥ 3). **(B)** Ligustilide attenuated cell migration in hypoxic TW2.6 cells. Cells were plated in 12-well plates overnight as a monolayer. A 200-μl tip was used to scratch a gap in the middle of the monolayers. Cells were washed with PBS and incubated with fresh serum-free medium with or without ligustilide and incubated at 37°C of hypoxia for 16 h. Cell migration was measured. Images are presented in 100 times magnification. Data in the plot were expressed as mean ± SEM (*n* ≥ 3). **(C)** Ligustilide reduced phospho-stat3 dose-dependently. TW2.6 cells were treated with ligustilide in different doses and harvested at 6 and 24 h of hypoxia. Protein was analyzed by Western blot. **P-valve < 0.01; ***P-valve < 0.001.

In the human oral cancer cell line TW2.6, the inhibitory effect of ligustilide was measured by MTT assay at 24 h of hypoxia. Cell viability was significantly reduced with 200 μM of ligustilide (**Figure 1A**). Pretreatment of the pan-caspase inhibitor Q-VD-OPh (10 μM) significantly rescued cell viability, a result that is also supported by cell images.

Cell migration affected by ligustilide was determined by the wound-healing assay. In our result, ligustilide decreased cell migration in TW2.6 cells in a dose-dependent manner at 16 h of hypoxia (**Figure 1B**).

Stat3 signaling is thought to play a critical role in promoting tumor survival and progression, including metastasis. We reported that phospho-stat3 in TW2.6 cells was augmented under hypoxia compared to normoxia. Ligustilide reduced phospho-stat3 levels at 6 h and 24 h of hypoxia in a dose-dependent manner (**Figure 1C**).

### Ligustilide Increased HIF-1α and c-Myc Protein Levels in Oral Cancer Cells

**Figure 2:**
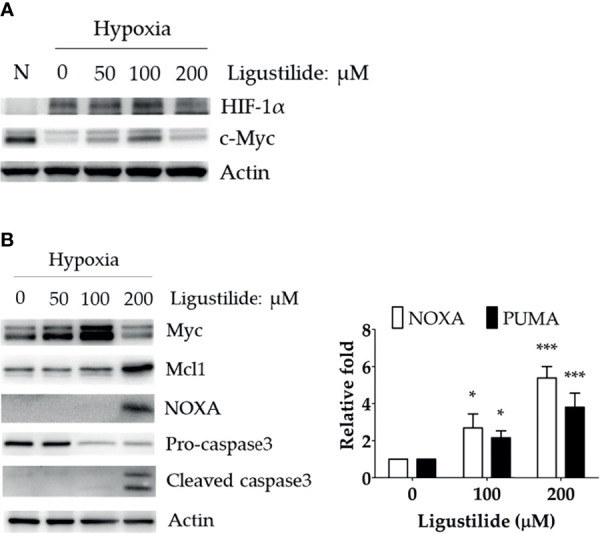
Effect of ligustilide on HIF-1α and c-Myc protein levels in hypoxic TW2.6 cells. **(A)** TW2.6 cells were treated with ligustilide and incubated at 37°C of hypoxia for 24 h. Cells were harvested and protein was analyzed by Western blot. A normoxia (N) group was performed as a control group compared to hypoxia. **(B)** Cells were treated with ligustilide in different doses and incubated at 37°C of hypoxia for 24 h. Protein (left) and mRNA (right) were harvested and analyzed. Data in the plot are expressed as mean ± SEM (*n* ≥ 3). *P-valve < 0.05; ***P-valve < 0.001.

We observed a significant increase in HIF-1α and c-Myc protein levels induced by ligustilide in a dose-dependent manner. Moreover, a sudden drop in both components was associated with a massive loss in cell viability (**Figure 2A**).

c-Myc induction was associated with an increase in pro-apoptotic proteins, including NOXA and cleaved caspase-3 (**Figure 2B**, left). In addition, the RNA levels of NOXA and PUMA were both enhanced by ligustilide (**Figure 2B**, right). It was noted that the anti-apoptotic protein Mcl-1, a direct target of c-Myc, increased to a peak level with 200 μM ligustilide. This observation suggests that c-Myc activity may be enhanced by ligustilide even when there is a sudden drop in its concentration at 200 μM ligustilide. Thus, upregulation of c-Myc was associated with ligustilide-induced apoptosis.

**Figure 3:**
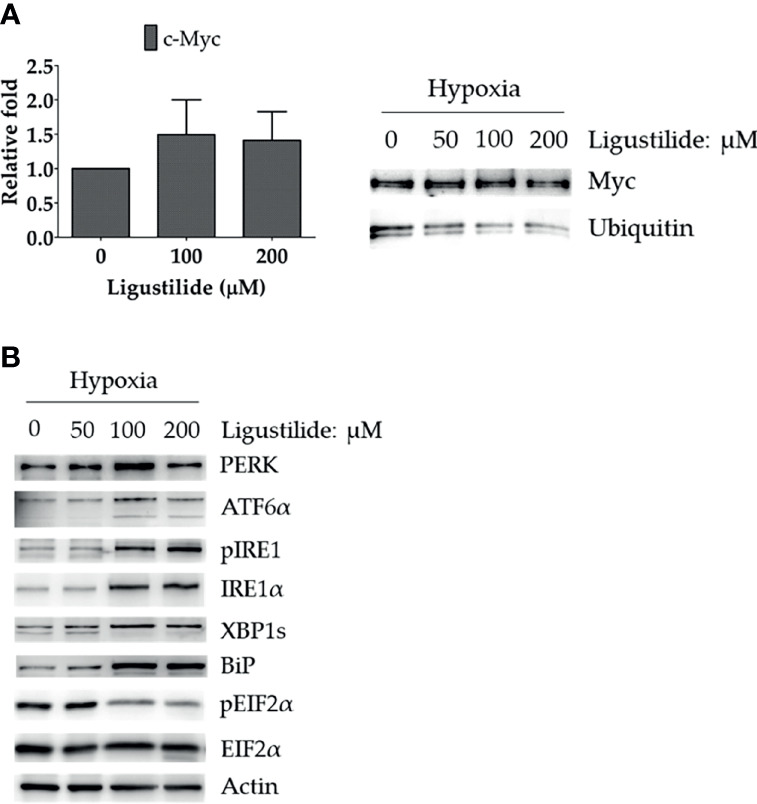
Effects of ligustilide on c-Myc mRNA and ubiquitinated c-Myc levels in hypoxic TW2.6 cells. **(A)** TW2.6 cells were treated with ligustilide and incubated for 24 h of hypoxia at 37°C. Messenger RNA and protein were harvested and c-Myc mRNA was measured (left). Ubiquitinated c-Myc levels were measured by immunoprecipitating c-Myc with anti-c-Myc antibody followed by detection with specific anti-ubiquitin or anti-c-Myc antibodies (right). Data in the plot are expressed as mean ± SEM (*n* ≥ 3). **(B)** Effects of ligustilide on ER stress-related signaling in hypoxic TW2.6 cells. Cells were treated with ligustilide in different doses and incubated at 37°C under hypoxia for 24 h. Protein was harvested and analyzed by Western blot.

To further investigate how c-Myc was increased by ligustilide, we determined the RNA level and found no significant alteration. However, ubiquitination of c-Myc protein was significantly attenuated by ligustilide as demonstrated by immunoprecipitation (**Figure 3A**). This result suggests that ligustilide increased the level of c-Myc *via* a post-translational pathway.

Furthermore, ER-stress signaling was decreased by ligustilide. PERK and ATF6α were induced by ligustilide, whereas PERK’s downstream target phosphorylation of EIF2α was inactivated (**Figure 3B**). Moreover, ligustilide elevated IRE1α in both total and phosphorylated levels, accompanied by the mediator BiP and downstream XBP1. These results indicate that ligustilide impaired ER-stress signaling in TW2.6 cells.

### Ligustilide-Induced c-Myc-Dependent Apoptosis *via* Activating ER-Stress Signaling

**Figure 4:**
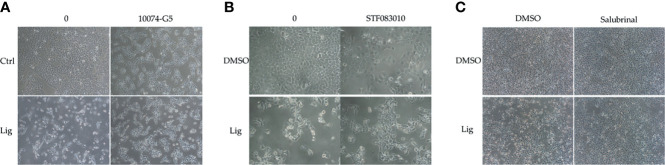
Effects of inhibitors on ligustilide-treated cells. TW2.6 cells were pretreated with inhibitors individually (**A**, 10074-G5: 5 μM; **B**, STF083010: 7.5 μM; **C**, Salubrinal: 40 μM) for 30 min and then with ligustilide (200 μM for group 10074-G5 and STF083010; 100 μM for group salubrinal). Images were taken at 24 h of hypoxia. Images for 10074-G5 are presented at 100 times magnification while those for STF083010 and salubrinal were at 200 and 60 times magnification, respectively.

We proposed that ligustilide induces c-Myc-dependent apoptosis. To support this hypothesis, we applied cells with the c-Myc inhibitor (10074-G5) prior to ligustilide. The cellular morphologies showed that co-treatment with 10074-G5 and ligustilide may restore cell numbers more than 2.85-fold compared to ligustilide alone (**Figure 4A** and **Supplementary Figure 1**). Similar effects were found in cells co-treated with IRE1 inhibitor (STF083010) and ligustilide compared to ligustilide alone (**Figure 4B**), and cell numbers were 1.87-fold higher. (**Supplementary Figure 1**). Moreover, cells treated with salubrinal, an agonist of phospho-EIF2α, showed a similar effect (**Figure 4C**).

**Figure 5:**
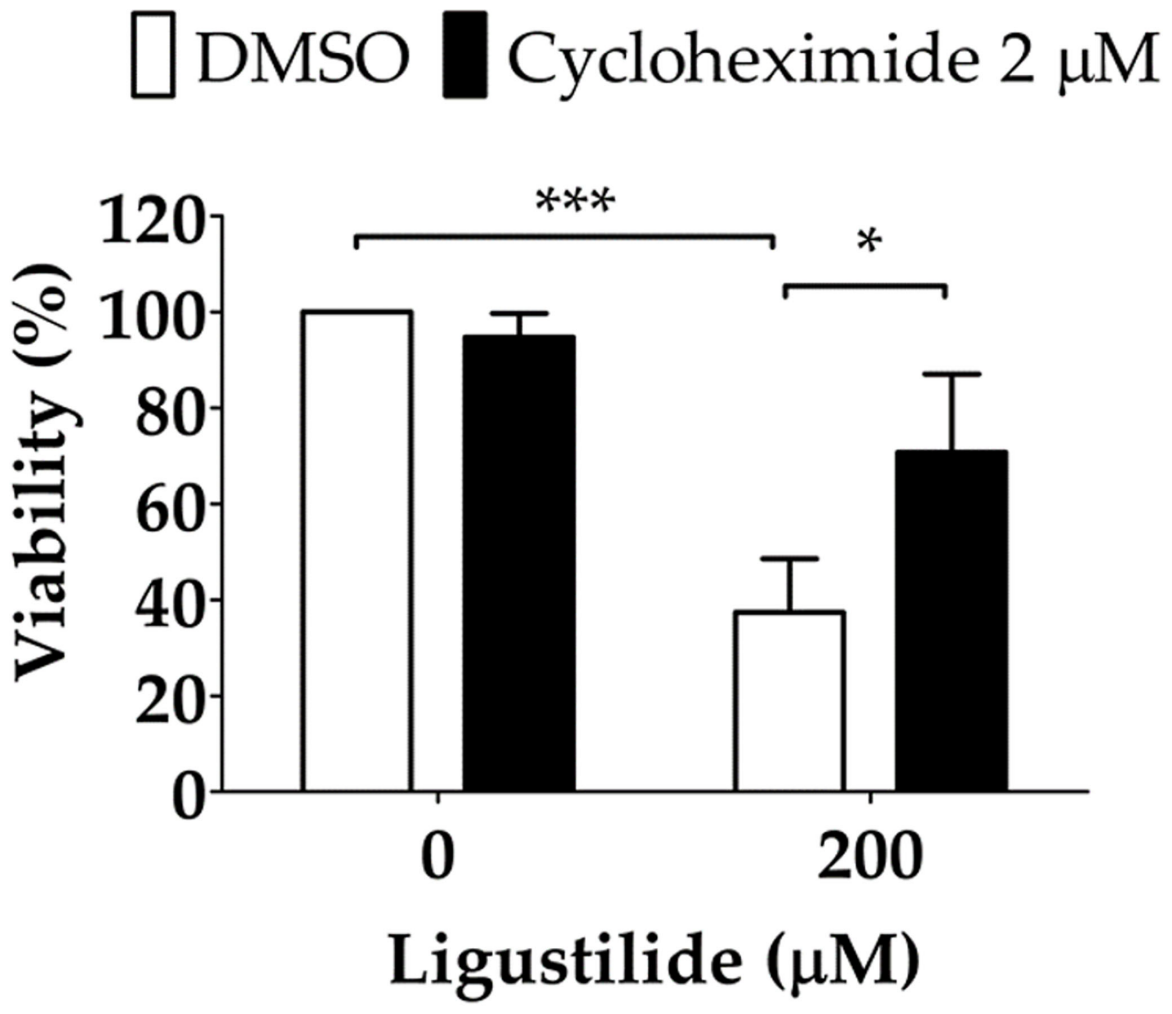
Effect of cycloheximide on ligustilide-induced cell death in hypoxic TW2.6 cells. Cells were pretreated with cycloheximide (2 μM) for 30 min and incubated with ligustilide for another 24 h under hypoxia. Cell viability was then measured by MTT assay. Data are expressed as mean ± SEM (*n* ≥ 3). *P-valve < 0.05; ***P-valve < 0.001.

It was noted that unfolded protein response attenuated global protein synthesis and activated certain factors that selectively promote protein translation to maintain proteostasis. Phosphorylation of EIF2α directly led to the inactivation of global protein synthesis. Thus, ligustilide decreased phospho-EIF2α, which could imply that cellular protein synthesis was augmented, and may lead to apoptosis. To support this hypothesis, the protein synthesis inhibitor cycloheximide was used. Cycloheximide (CHX) can be used as a ribosome inhibitor as well as an ER-stress inhibitor. Therefore, CHX increased cell viability in ligustilide-treated cells, which may represent ligustilide-induced ER stress, and further induce cell death. We found that cell viability was significantly restored in cells co-treated with cycloheximide and ligustilide compared to ligustilide alone (**Figure 5**).

### Ligustilide Enhanced the Radiosensitivity of Oral Cancer

**Figure 6:**
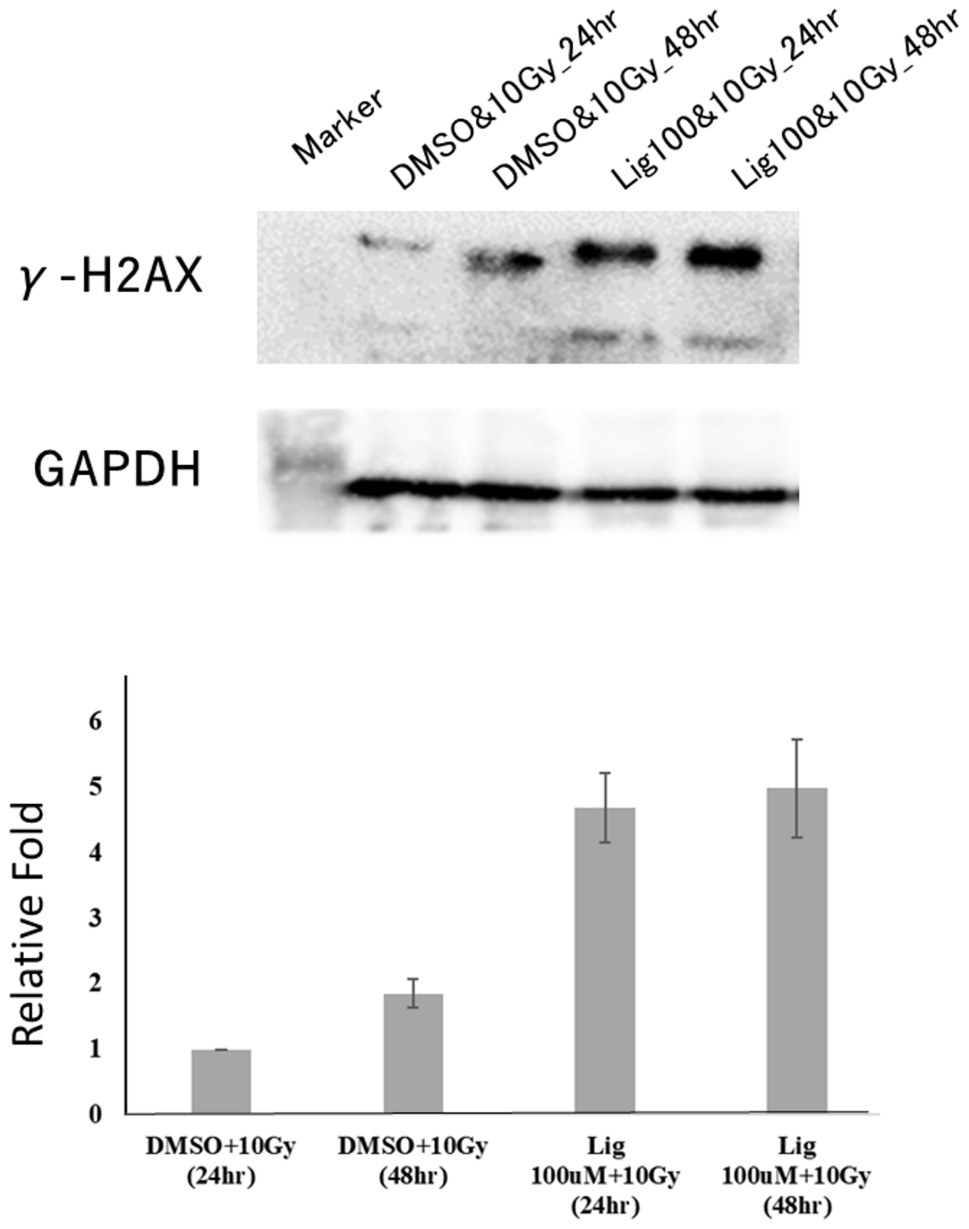
Ligustilide can increase the expression of γ-H2AX and enhance the occurrence of DNA damage in oral cancer cells. After treating oral cancer cells with ligustilide (100 μM) for 24 h, the cancer cells received a dose of 10 Gy. The cancer cells treated with ligustilide and 10 Gy produced a large amount of γ-H2AX protein after 24 h and its expression even exceeded the control group treated with DMSO and 10 Gy for 48 h. GAPDH is internal control. The blots were representative of three independent experiments. Data were expressed as mean ± SD (*n* ≥ 3).

Studies have found that ligustilide can enhance the DNA damage caused by tamoxifen to further enhance the sensitivity of breast cancer cells to drugs (8). Therefore, we hypothesized whether ligustilide could be used as a radiosensitizer to increase radiation damage to the DNA of cancer cells. After oral cancer cells were treated with ligustilide for 24 h, they received a 10-Gy dose of radiation. The results showed that the expression of γ-H2AX in cancer cells treated with ligustilide was higher than that in the control group (DMSO treatment) at both 24 h and 48 h after irradiation (**Figure 6**).

## Discussion

c-Myc degradation under hypoxia is well-known in cancer cells as a means of surviving in conditions of low oxygen tension (24). In the present study, we reported that in hypoxic TW2.6 cells, c-Myc level did recapitulate this phenomenon (**Figure 4**). Ligustilide increased c-Myc levels, however, a change associated with cell death, which gives us reason to hypothesize that ligustilide-induced cell death is mediated by c-Myc upregulation. Co-treatment with c-Myc inhibitor 10074-G5 may rescue cells, suggesting that c-Myc-dependent apoptosis might be involved (**Figures 4A, B**).

Proteasome inhibitors are effective candidates for cancer therapies targeting the ubiquitin–proteasome pathway (UPP or UPS) (25). It has been shown that colon cancer cells, in response to the proteasome inhibitor bortezomib, may initiate cell death by elevating the expression of the BH3-only members NOXA and BIM. It was further elucidated that accumulation of c-Myc by bortezomib may drive this process (26). Nevertheless, an inhibitor of Nedd8-activating enzyme, MLN4924, was shown to augment c-Myc and induce apoptosis by elevating NOXA in leukemia cells (27). Overall, proteasomal inhibition of c-Myc degradation may result in BH3-only protein-dependent apoptosis in cancer. Accordingly, we propose that ligustilide exerts inhibitory activity on c-Myc degradation and leads to apoptosis (**Figure 3A**).

EIF2α receives signals from the UPR sensors in response to stress and attenuates global protein synthesis through phosphorylation. We found that ligustilide significantly blocked phospho-EIF2α in a dose-dependent manner. This result could imply that elevation of protein synthesis might be required for ligustilide-induced apoptosis. The ribosome inhibitor cycloheximide or the phospho-EIF2α agonist salubrinal promoted the restoration of either cell viability or cell numbers and improvements in cellular morphology when co-treated with ligustilide, which supports the hypothesis that protein synthesis or phospho-EIF2α may play a role in ligustilide-induced apoptosis. Nonetheless, IRE1α, along with the phosphorylation increased by ligustilide, is associated with ligustilide-induced cell death, which could be compromised by the IRE1α inhibitor STF083010. Overall, the role of IRE1α signaling in ligustilide-induced apoptosis requires further investigation.

HIF-1α is critical for cellular adaption under hypoxic conditions and is often associated with tumor progression and survival. We found that the elevation of HIF-1α was associated with ligustilide-induced apoptosis, although a sudden decline occurred, similar to that in the level of c-Myc. Although some evidence suggests that IRE1α may promote HIF-1 activity in breast cancer cells (28), the increased HIF-1α in our study could be explained by the inhibition of proteasome activity as we hypothesized. However, proof of the exact reason for this or the role of HIF-1α in response to ligustilide requires further experimental evidence.

Constitutive activation of stat3 signaling is common in cancer and usually correlates with poor survival rate in patients and tumor resistance to drugs. We have reported that phospho-stat3 was reduced by ligustilide in a dose-dependent manner. This observation could imply that inhibition of stat3 signaling by ligustilide might directly or indirectly affect c-Myc-induced apoptosis as we proposed. Consequently, we may not be able to obtain a dramatic reversion when performing rescue experiments because attenuation of phospho-stat3 is not restored. However, it is difficult to identify the component that directly causes stat3 inactivation by ligustilide because there are numerous candidates that need to be individually identified with the aid of RNAi. Importantly, since we have observed significant changes in cells co-treated with ligustilide plus inhibitors compared to ligustilide alone, c-Myc-dependent apoptosis induced by ligustilide may be independent on stat3, at least in part.

Surprisingly, Western blot results showed that a higher expression of γ-H2AX was observed after treatment with ligustilide compared to the control group. Since γ-H2AX is a mature DSB marker, this result indicates that cells treated with ligustilide are more susceptible to radiation, which increases the effect of DNA damage in cancer cells. However, this is the first time that ligustilide has been shown to have potential as a radiation sensitizer. Ligustilide may cause this effect by changing Histon’s expression in epigenetics (**Supplementary Table 1**), but additional experiments are needed to identify the relevant molecular mechanism.

**Figure 7:**
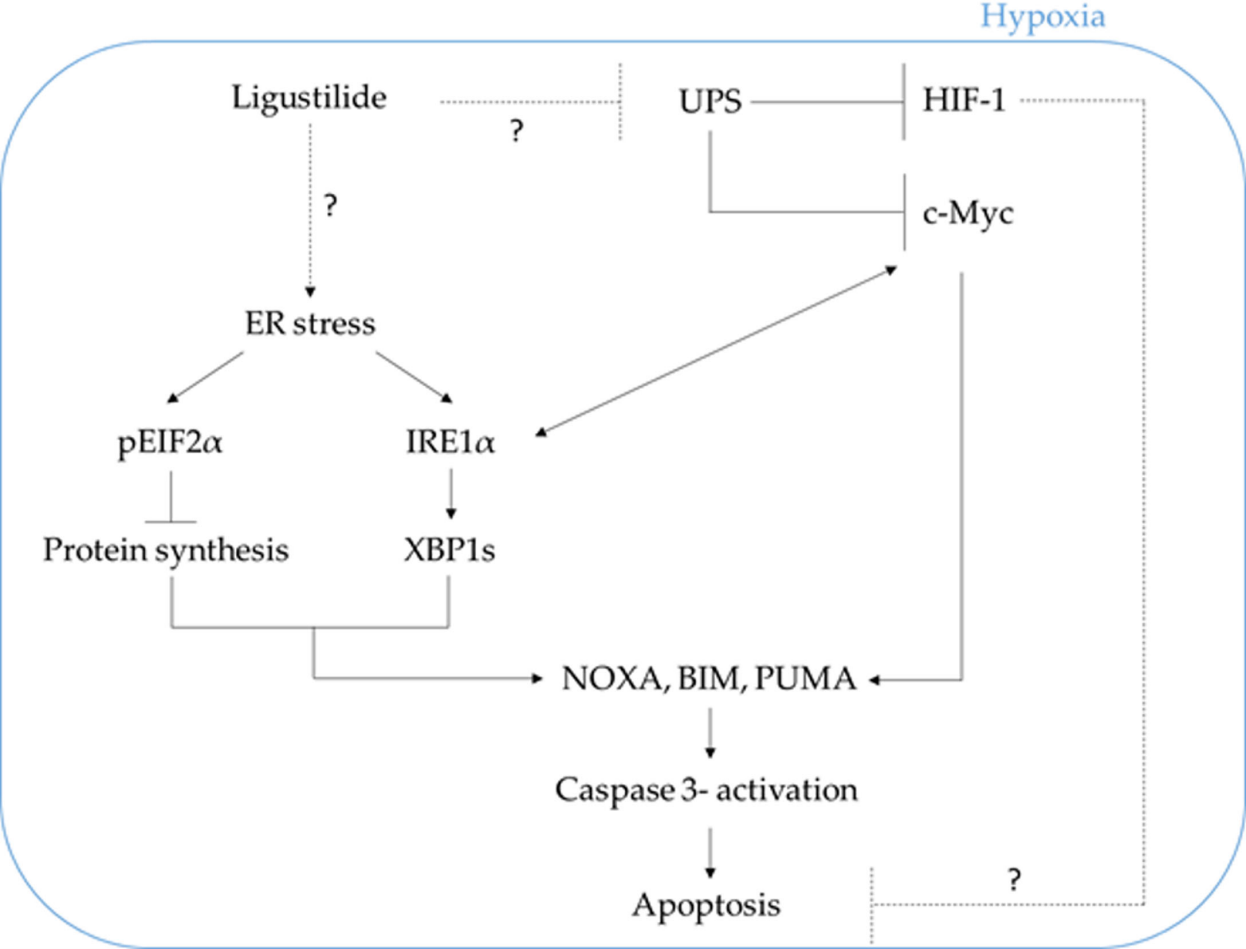
The schematic plot illustrates the proposed pathway in which ligustilide-induced apoptosis in hypoxic oral cancer cells is dependent on c-Myc upregulation resulting from inhibition of proteasome degradation and elevation of ER-stress signaling. The role of HIF-1 in this system is not clear and awaits additional experimental evidence.

We propose that ligustilide induces c-Myc-dependent apoptosis by activating ER-stress signaling in hypoxic oral cancer cell lines including TW2.6 and OML1 (**Figure 7**).

## Data Availability Statement

The original contributions presented in the study are included in the article/**Supplementary Material**. Further inquiries can be directed to the corresponding author.

## Author Contributions

D-WL, K-YP, and R-JH thought of the concept and designed the study. Y-TC provided statistical analysis. W-LH provided study materials. All authors collected and assembled data, conducted data analysis, wrote, and made final approval of the manuscript.

## Funding

This work was financially supported by the Hualien Tzu Chi Hospital, Buddhist Tzu Chi Medical Foundation in Taiwan, under Grant TCRD110-046, TCRD111-067, and TCRD111-075.

## Conflict of Interest

The authors declare that the research was conducted in the absence of any commercial or financial relationships that could be construed as a potential conflict of interest.

## Publisher’s Note

All claims expressed in this article are solely those of the authors and do not necessarily represent those of their affiliated organizations, or those of the publisher, the editors and the reviewers. Any product that may be evaluated in this article, or claim that may be made by its manufacturer, is not guaranteed or endorsed by the publisher.
