# Supplementary material for: Z-Ligustilide Induces c-Myc-Dependent Apoptosis via Activation of ER-Stress Signaling in Hypoxic Oral Cancer Cells
Source: Front Oncol. 2022 Apr 13;12:824043. doi: 10.3389/fonc.2022.824043 (PMC9043595; doi:10.3389/fonc.2022.824043)
Supplement: Supplementary file 1 [file Presentation_1.pptx]

## Slide 1
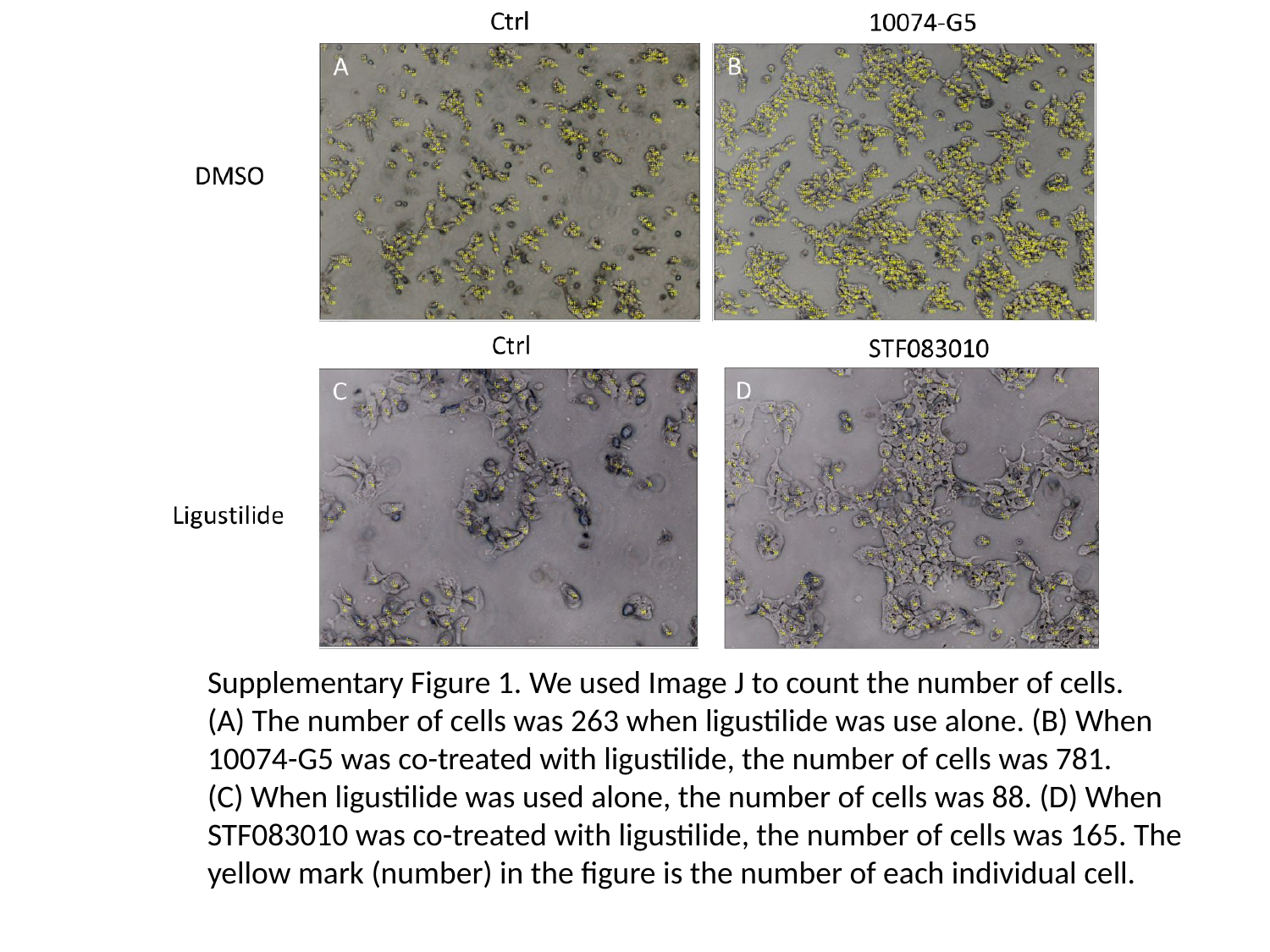

B
A
B
Supplementary Figure 1. We used Image J to count the number of cells.
(A) The number of cells was 263 when ligustilide was use alone. (B) When 10074-G5 was co-treated with ligustilide, the number of cells was 781.
(C) When ligustilide was used alone, the number of cells was 88. (D) When STF083010 was co-treated with ligustilide, the number of cells was 165. The yellow mark (number) in the figure is the number of each individual cell.
