# Supplementary material for: Z-Ligustilide Induces c-Myc-Dependent Apoptosis via Activation of ER-Stress Signaling in Hypoxic Oral Cancer Cells
Source: Front Oncol. 2022 Apr 13;12:824043. doi: 10.3389/fonc.2022.824043 (PMC9043595; doi:10.3389/fonc.2022.824043)
Supplement: Supplementary file 2 [file Table_1.pdf]

Supplementary Table 1. Differential Type of Histones are Overexpression in High Grade

Gene	FDR	Gene	FDR	Gene	FDR	Gene	FDR	Gene	FDR
**H2AC4**	2.46E-25	**H2BC3**	2.89E-16	**H3C1**	3.24E-13	**H4C1**	5.63E-33	**CCNA2**	1.38E-25
**H2AC7**	4.70E-20	**H2BC6**	2.04E-22	**H3C4**	1.21E-14	**H4C9**	3.51E-19	**ETV7**	2.04E-12
**H2AC11**	6.10E-32	**H2BC9**	1.00E-22	**H3C7**	4.98E-32	**H4C11**	1.34E-23	**MMP9**	4.34E-15
**H2AC12**	1.22E-24	**H2BC10**	6.79E-18	**H3C8**	2.64E-10	**H4C12**	3.72E-24		
**H2AC13**	4.55E-39	**H2BC11**	3.32E-25	**H3C10**	2.78E-37	**H4C14**	1.20E-37		
**H2AC14**	3.02E-34	**H2BC12**	1.47E-27	**H3C11**	7.65E-23	**H4C15**	3.72E-37		
**H2AC16**	1.29E-33	**H2BC13**	3.94E-34	**H3C12**	3.29E-26				
**H2AC17**	2.65E-38	**H2BC14**	3.66E-23	**H3C13**	3.34E-26				
**H2AC18**	1.23E-34	**H2BC17**	1.51E-39	**H3C14**	1.31E-43				
**H2AC19**	1.02E-39	**H2BU1**	3.35E-19	**H3C15**	1.31E-43				
**H2AC21**	2.23E-25								
**H2AW**	1.63E-17								
**H2AX**	7.89E-33								
**H2AZ1**	1.15E-32								
